# Cystic pancreatic lesions: MR imaging findings and management

**DOI:** 10.1186/s13244-021-01060-z

**Published:** 2021-08-10

**Authors:** Giovanni Morana, Pierluigi Ciet, Silvia Venturini

**Affiliations:** 1 Department of Radiology, Regional Hospital Ca’ Foncello, Piazza Ospedale 1, 3100 Treviso, Italy; 2 Department of Radiology and Nuclear Medicine, Erasmus MC, Rotterdam, The Netherlands

**Keywords:** Pancreatic cyst, Pancreatic neoplasms, Pancreatic intraductal neoplasms, Cystadenoma, Serous, Cystadenoma, Mucinous

## Abstract

Cystic pancreatic lesions (CPLs) are frequently casual findings in radiological examinations performed for other reasons in patients with unrelated symptoms. As they require different management according to their histological nature, differential diagnosis is essential. Radiologist plays a key role in the diagnosis and management of these lesions as imaging is able to correctly characterize most of them and thus address to a correct management. The first step for a correct characterization is to look for a communication between the CPLs and the main pancreatic duct, and then, it is essential to evaluate the morphology of the lesions. Age, sex and a history of previous pancreatic pathologies are important information to be used in the differential diagnosis. As some CPLs with different pathologic backgrounds can show the same morphological findings, differential diagnosis can be difficult, and thus, the final diagnosis can require other techniques, such as endoscopic ultrasound, endoscopic ultrasound-fine needle aspiration and endoscopic ultrasound-through the needle biopsy, and multidisciplinary management is important for a correct management.

## Key points

- CPLs are frequently detected lesions with different malignant potential, requiring different management.
- MRI with MRCP best allows detection of those imaging features helpful for lesion characterization.
- A CPL communicating with MPD is an IPMN.
- A CPL non-communicating with MPD is related to different pathologies (SCN, SPN, MCN).
- Differential diagnosis can be done using morphological appearance, location and demographic data.

## Introduction

Cystic pancreatic lesions (CPLs) are quite common: Their frequency of detection ranges from 2.4 to 19.6%, and their prevalence as well as size and number increases with age (from 7.9 below 70 years to 40.2 over 70 years) [1–5]. A precise characterization is fundamental for the correct management of these lesions, as they have heterogeneous biological behavior and different prognosis (according to histological type and differentiation), thus requiring different therapeutic options [6].

However, difficulties in differential diagnosis of CPLs still exist because of the lack of specific clinical and laboratoristic signs and the overlap of imaging findings, and thus, the management of patients with CPLs remains complex [7]. Moreover, the frequent incidental detection of CPLs, in the absence of any symptoms, makes the diagnosis even more difficult [8].

Most CPLs can be considered “technopathies,” as they are more frequently detected in the last decades due to the widespread use and advancement in diagnostic imaging. The vast majority of these lesions will never threat the life of affected subjects, but due to their malignant potential will cause affected subjects to become patients, and followed up even for many years. Follow-up strategies rise a really challenging issue for radiologists, due to the high number of patients to be submitted. The role of Imaging is to differentiate benign from malignant or potentially malignant CPLs avoiding unnecessary surgery and, in potentially malignant CPLs, to early detect morphological changes related to malignant transformations in order to offer more chance of survival to these patients.

Scope of this review is to offer a practical approach to the diagnosis of CPLs using mainly MR imaging findings, location and demographic data and thus drive their correct management. Differential diagnosis among different CPLs is also emphasized.

### Classification

According to data extrapolated from WHO classification, CPLs are classified in epithelial and non-epithelial and each of these categories is further subdivided in non-neoplastic and neoplastic (CPNs) (Table 1) [9]

**Table 1:** Classification of cystic pancreatic lesions (CPLs)

WHO classification of cystic pancreatic lesions	
**Epithelial non-neoplastic**	**Epithelial neoplastic**
	*Mucinous*
	Intraductal papillary mucinous neoplasm
	Mucinous cystic neoplasm
Lymphoepithelial cyst	
Mucinous non-neoplastic cyst	*Non-mucinous*
Enterogeneous cyst	Serous cystic neoplasm
Retention cyst/dysontogenetic cyst	Solid pseudopapillary neoplasm
Periampullary duodenal wall cyst	Cystic neuroendocrine tumor G1-2
Endometrial cyst	Acinar cell cystadenoma
Congenital cyst (in malformation syndromes)	Serous cystoadenocarcinoma
	Cystic ductal adenocarcinoma
	Cystic acinar cell carcinoma
	Accessory-splenic epidermoid cyst
	Cystic hamartoma
	Cystic teratoma
	Cystic pancreatoblastomas
	Cystic metastatic epithelial neoplasm
	Others
**Non-epithelial non-neoplastic**	**Non-epithelial neoplastic**
Walled-off necrosis	Benign non-epithelial neoplasms (e.g., lymphangioma)
Pancreatitis-associated pseudocyst	
Parasitic cyst	Malignant non-epithelial neoplasms (e.g., sarcomas)

Epithelial CPNs are further divided in mucinous, always premalignant–malignant lesions and non-mucinous neoplasms, which include both benign (most frequent) and borderline and malignant lesions.

In particular, mucinous CPNs include mucinous cystic neoplasm (MCN) and intraductal papillary mucinous neoplasm (IPMN), while non-mucinous CPNs include serous cystic neoplasm (SCN), solid pseudopapillary neoplasm (SPN), cystic neuroendocrine neoplasm (CPNET), the rarer acinar cell cystic neoplasm (ACCN), ductal adenocarcinoma with cystic degeneration and other rarer lesions (Table 1).

Many others CPLs can be included in this review, but due to their very low incidence, their discussion will be omitted [10].

From the radiological viewpoint, the key factor to characterize a CPL is to establish whether the lesion communicates or not with the main pancreatic duct (MPD) (Table 2).

**Table 2:** Radiological classification of cystic pancreatic lesions (CPLs)

Cystic pancreatic lesions: radiological classification	
**Non-communicating with main pancreatic duct**	
Non-neoplastic	Walled-off necrosis
	Congenital cyst
	Retention Cyst
Neoplastic	Mucinous
	Mucinous cystic neoplasms
	Non-mucinous
	Serous cystic neoplasm
	Solid pseudopapillary neoplasm
	Cystic pancreatic neuroendocrine neoplasm
	Acinar cell cystic neoplasm
	Ductal adenocarcinoma with cystic degeneration
**Communicating with main pancreatic duct**	
Non-neoplastic	Pseudocyst
	Walled-off necrosis
Neoplastic	Intraductal papillary mucinous neoplasm

CPLs non-communicating with MPD include non-neoplastic (walled-off necrosis—WON, simple or congenital cyst and retention cyst) as well as neoplastic cysts [11, 12]. Neoplastic non-communicating cysts include mucinous (MCN) and non-mucinous neoplasms (SCN, SPN, ACCN, CPNET and ductal adenocarcinoma with cystic degeneration). CPLs communicating with MPD are further divided in non-neoplastic (pseudocyst and WON) and neoplastic (IPMN).

### Imaging techniques

Nowadays, CPLs can be detected with most imaging techniques (ultrasound—US, multidetector computed tomography—MDCT, magnetic resonance imaging with magnetic resonance cholangiopancreatography—MRI with MRCP), but for a correct characterization MDCT and MRI are needed. Endoscopic ultrasound—EUS—is another useful imaging technique to correctly characterize CPLs, but its use is mostly managed by gastroenterologists, and thus, its description is beyond the scope of this paper.

MRI with MRCP, thanks to its high contrast resolution and high sensitivity to static fluids on T 2 w sequences, is the best imaging technique to assess communication with MPD (which is the key factor to characterize a CPL); on the other hand, MDCT is the best imaging technique to demonstrate the presence, the intra-lesional localization and the size of eventual calcifications, helpful findings in the differential diagnosis; moreover, in elderly and uncooperative patients MDCT with curved multiplanar reconstruction (MPR) post-processing is a valid technique to assess communication with MPD. Both MRI with MRCP and MDCT have high diagnostic performance in differentiating benign from malignant CPLs, with an accuracy ranging from 73 to 81% for MRI and 75% to 78% for MDCT, respectively [13–16].

### CPLs non-communicating with MPD: non-neoplastic

#### Pseudocyst

Pancreatic pseudocyst is a pancreatic and/or peripancreatic fluid collection with well-defined walls containing pancreatic juice or amylase-rich fluid and essentially no solid material. It is considered a delayed (usually > 4 weeks) complication of interstitial edematous pancreatitis [11, 17].

**Fig. 1:**
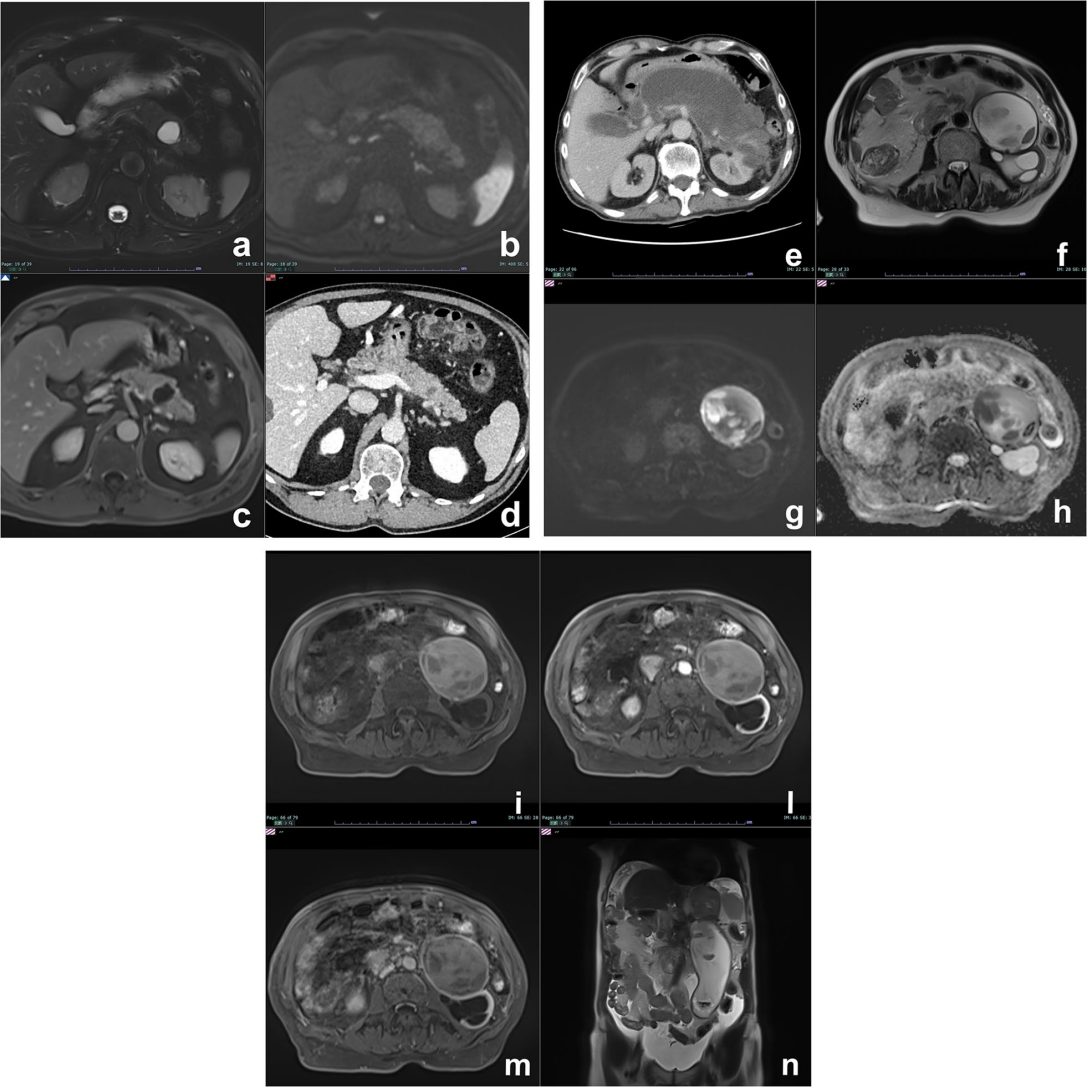
Cystic complications of acute pancreatitis. **a**–**d** Pseudocyst. Patient with a previous interstitial edematous pancreatitis. At follow-up after 1 year, a cystic lesion with no septa or debris is appreciable in the tail of the pancreas, hyperintense on T2w (**a**), without high signal intensity on high b value DWI (**b**) and no enhancement after contrast media administration (**c**). At further follow-up after 6 months, the lesion is decreased in size (**d**). **e**–**n**: WON. A large necrotic collection is appreciable in a patient with necrotizing acute pancreatitis (**e**). After 1 year, a large collection with debris is still appreciable in the tail of the pancreas, clearly visible on T2w as cystic lesion with low signal intensity foci (**f**). Debris show inhomogeneous signal intensity on DWI (**g**), ADC map (**h**) and T1 unenhanced (**i**), but do not show any enhancement after contrast media administration (**l**, **m**). On T2 HASTE coronal view, the caudal extension of the collection is appreciable (**n**)

At MRI, pseudocyst is markedly hyperintense on T2w and hypointense on T1w (Fig. 1), very similar to other pancreatic cysts so to make confident diagnosis of pseudocyst, and it is necessary to have correlation with clinical history of acute pancreatitis [18]. Cui et al. in a multicenter study demonstrated that more than 80% of pseudocysts disappeared or decreased in size during follow-up (Fig. 1d) [19].

However, acute pancreatitis can be caused by cystic tumors (IPMN), or patients with acute pancreatitis may have cystic tumors, so a previous acute pancreatitis cannot completely exclude the presence of a true cystic tumor.

#### WON

WON is an encapsulated collection of pancreatic and/or peripancreatic necrosis surrounded by enhancing walls of reactive tissue that occurs ≥ 4 weeks after onset of necrotizing pancreatitis as result of the organization of an acute necrotic collection [17]. WON contains necrotic fat and/or pancreatic tissue which manifest at MRI as intracystic non-liquefied debris, an highly specific MR finding for the diagnosis of WON (Fig. 1e–n) [11, 20].

#### Congenital cyst

Congenital cyst or “true cyst” is an extremely rare cystic lesion lined by a single layer of cuboidal epithelium; it mostly occurs in children or in patients with polycystic disorders (e.g., autosomal dominant polycystic kidney disease) and is usually mistaken or finally diagnosticated by exclusion [21].

#### Retention cyst

Retention cyst is a cystic dilatation of a pancreatic duct, usually caused by calculi, mucin, chronic pancreatitis or pancreatic cancer.

**Fig. 2:**
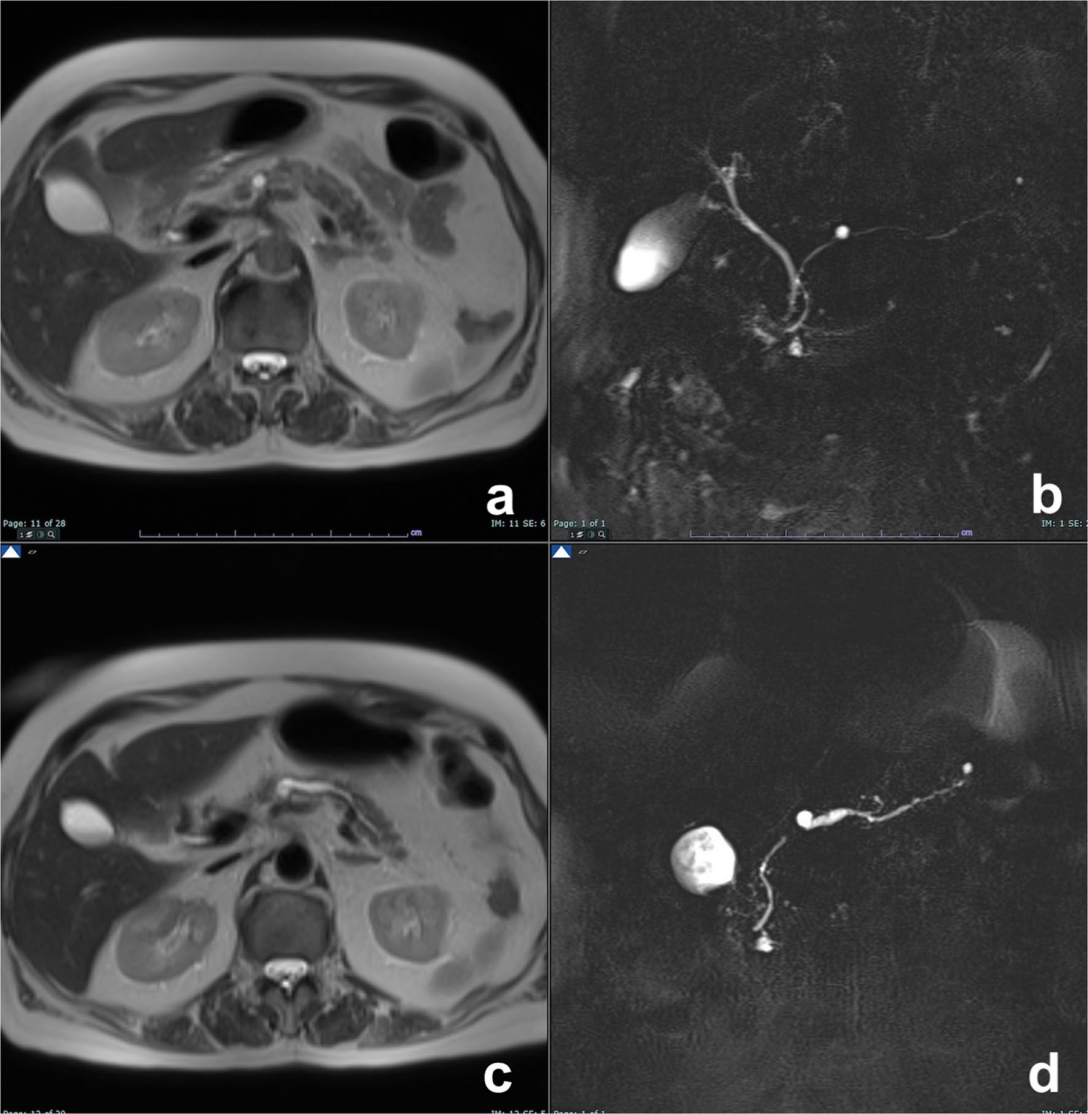
Retention cyst. A small cyst is observed on T2w (**a**) and MRCP (**b**), without evidence of a mass. On a follow-up study two years later, a focal obstruction of the main pancreatic duct appeared with chronic obstructive pancreatitis features upstream (**c**, **d**), all findings of high suspicion of malignancy (pancreatic carcinoma on histology). (From: Morana G, Faccinetto A, Venturini S: Pancreas. In: Vanzulli A, Colagrande S, Grazioli L, Morana G (eds), MRI of the abdomen—technique and imaging findings. Poletto, Milan, 2021)

Retention cyst has mucinous mucosal lining and has imaging features similar to those of small MCN or of BD-IPMN [22]. As it can be the first sign of a pancreatic cancer, even if small, it cannot be underestimated and must undergo follow-up (Fig. 2), particularly in young or middle-aged patients [23].

As retention cyst has no characteristic imaging features, it must be considered an indeterminate cyst whose management is different according to different guidelines, based on only the size [9] or balanced on the size and the age of the patient [24]. As a role of thumb, in very small lesions (< 5 mm) a stability after 2-year follow-up is sufficient to stop surveillance [24]; in larger cysts in young (< 65 yo) and fit patients, follow-up by imaging should last at least 9–10 years if the lesion is stable [24], while for other guidelines there are not clear indications on the length of follow-up [9].

### CPL non-communicating with MPD: neoplastic

The most common cystic pancreatic neoplasms non-communicating with MPD are SCN, MCN, SPN, CPNET and ACCN, and these entities can be distinguished on the basis of their morphological features, location and demographic data.

#### Serous cystic neoplasm (SCN)

SCN is a benign lesions, and only very few cases of malignant degeneration have been published [25]. It accounts for 10–15% of CPLs and for around 1–2% of all pancreatic neoplasms [26, 27].

It is composed by multiple cysts formed by glycogen-rich epithelial cells and mainly involves the head of the pancreas, although it can be located in any part of the gland [28].

It primarily affects females in their 50 s [26], but it is often detected later.

**Fig. 3:**
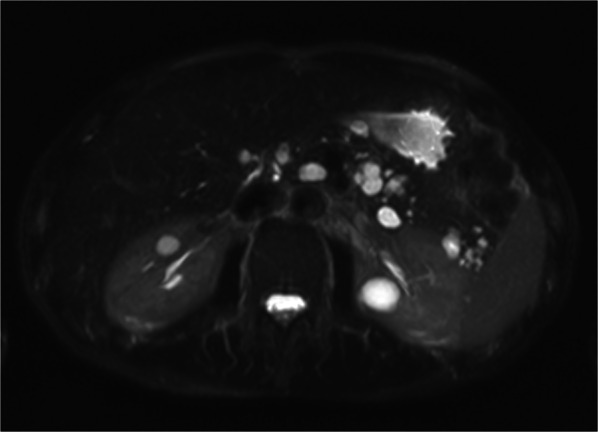
Von Hippel–Lindau. Several cysts are visible on T2w: multifocal SCN

On 15–30% of cases, SCN is associated with von Hippel–Lindau disease (VHL) and in these cases tends to be multifocal and may involve diffusely the pancreatic gland [29] (Fig. 3).

SCN is mostly (nearly 60%) asymptomatic and only when large can cause signs and symptoms of obstructive chronic pancreatitis such as unspecific abdominal pain, diabetes mellitus and pancreaticobiliary symptoms [26].

At imaging, SCN may manifest with three main patterns, reflecting its morphological appearance: microcystic (or polycystic), honeycomb and macrocystic (or oligocystic) [30].

On a multinational review on 2622 patients with SCA [26], authors observed that the most frequent pattern was microcystic pattern (45%), followed by macrocystic pattern (32%), mixed type (18%) and honeycomb pattern (5%).

**Fig. 4:**
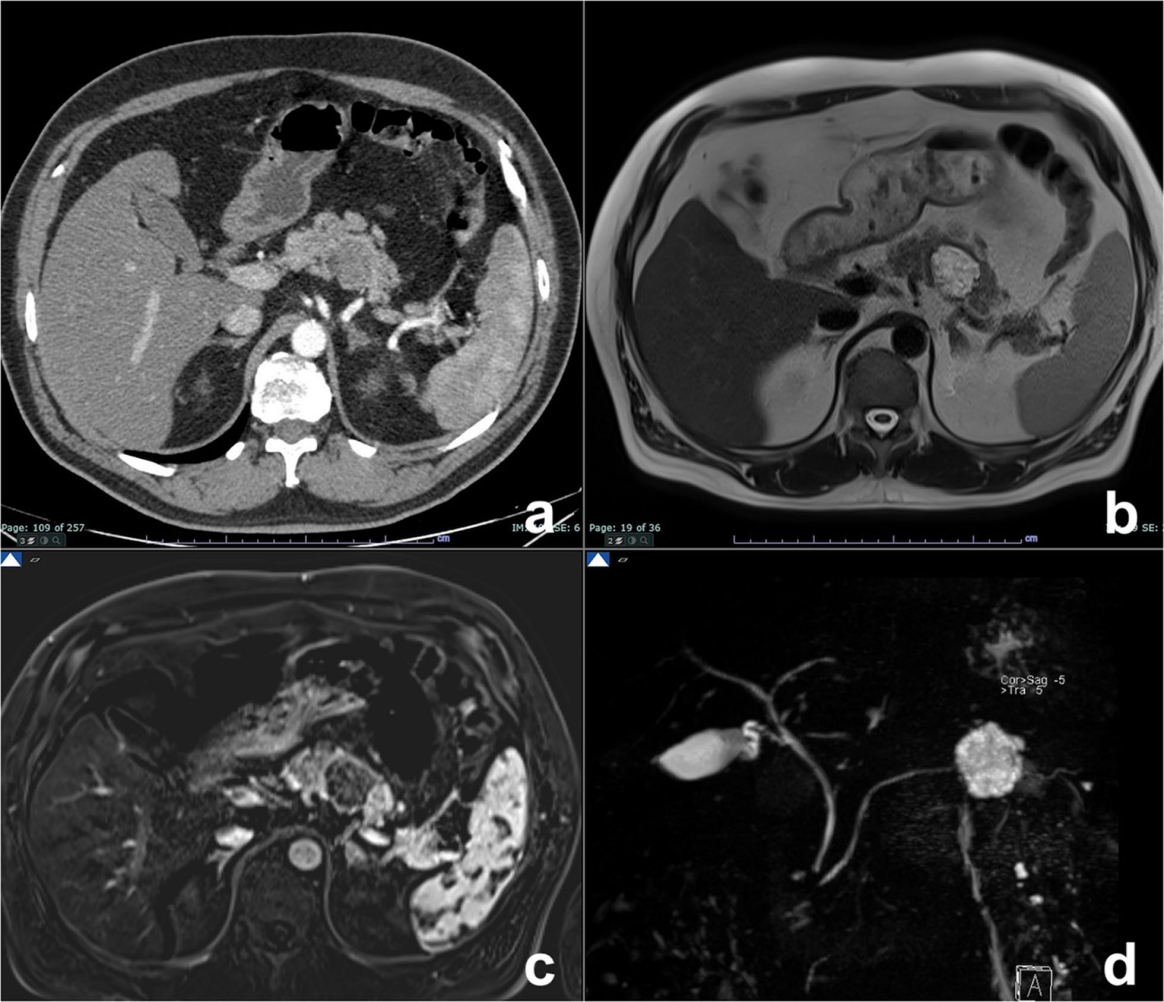
Serous cystadenoma, “honeycomb” pattern. On contrast enhanced CT (**a**), a large hypodense lesion with tiny central enhancement can be appreciated in the body–tail of the pancreas. On T2w MRI, the lesion is hyperintense (**b**). On T1w dynamic imaging, the central enhancement is appreciable, especially with subtraction imaging (**c**), related to the enhancement of septa and stroma supporting the microcysts. MRCP (**d**)

The more common microcystic pattern is composed by multiple cysts < 2 cm separated by fibrous septa that can converge into a central stellate scar that may calcify. In the rarer mixed type, few larger cysts (> 2 cm) are located peripherally. When cysts are numerous and subcentimeters, the lesion assumes an “honeycomb” pattern (Fig. 4) [30].

Some authors observed that central scar had sensitivity (Se), specificity (Sp) and positive predictive value (PPV) of 32.4%, 100% and 100%, respectively, for diagnosis of SCA, while combination of microcystic appearance and lobulated margins had Se, Sp and PPV, respectively, of 68%, 100% and 100% [31], suggesting that when present, both central scar and combination of microcystic appearance and lobulated margins are pathognomonic of SCN.

Some other authors observed that on MDCT, also the presence of a central calcification and the “circumvascular sign” (i.e., presence of some abnormal arteries surrounding the lesion on arterial phase) is pathognomonic for SCN (100% Sp), and however, central calcification has a very low sensitivity (less than 30%), while circumvascular sign has a Se of 76.7% [32].

**Fig. 5:**
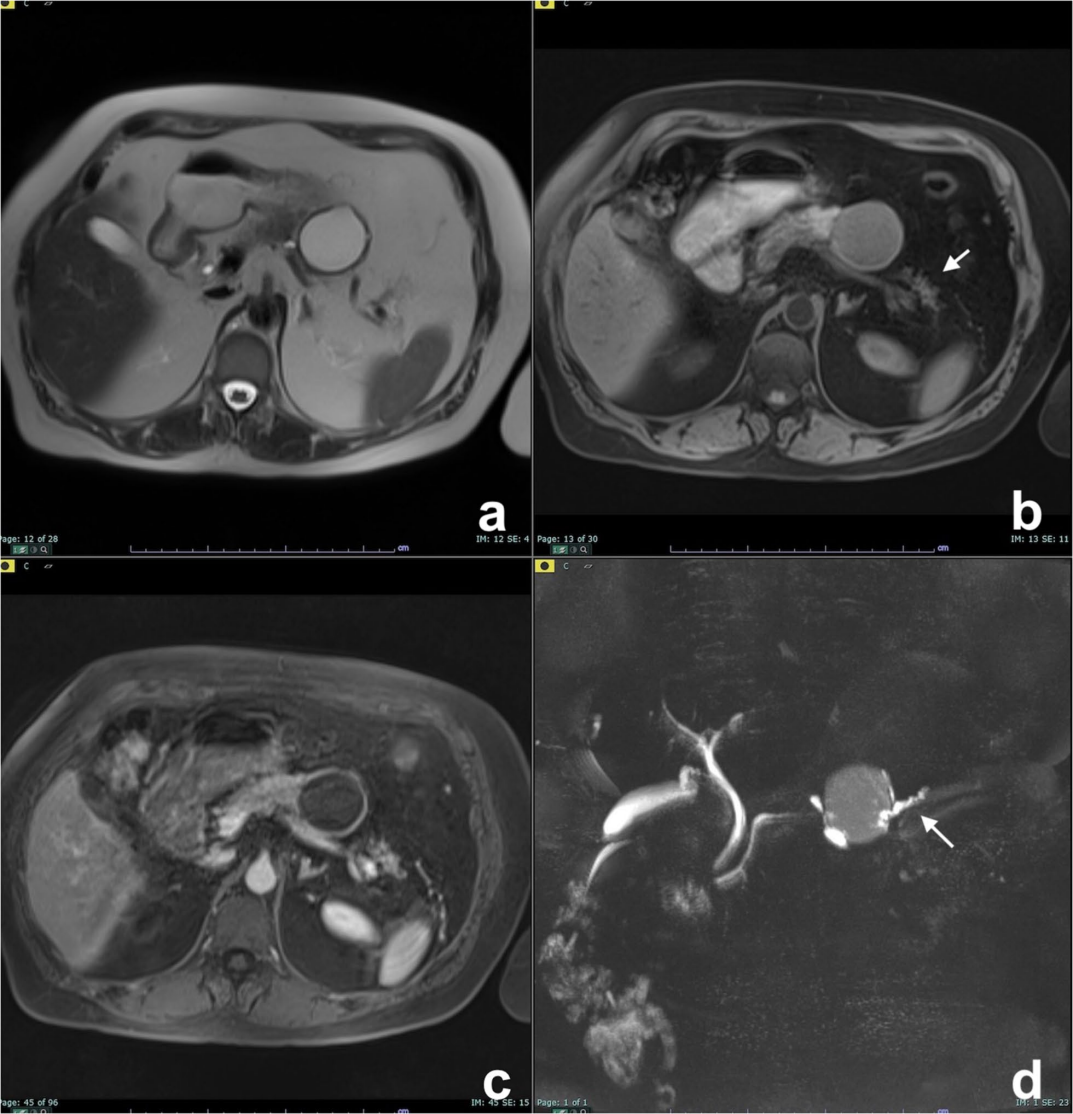
Serous cystadenoma, macrocystic pattern. Female, 57 yo. A large cist with thick walls is visible in the body of the pancreas with no nodules or enhancing septa both on T2w (**a**) and T1w before (**b**) and after contrast media administration (**c**) with atrophy of the tail (arrow in **b**). At MRCP (**d**), a dilatation of the MPD upstream is recognizable (arrow). Due to the high suspicious of a mucinous cystadenoma, the lesion was resected, and at pathology, a serous cystoadenoma was diagnosed

The macrocystic pattern (Fig. 5) is formed by a small number of cysts ≥ 2 cm without a central scar, and is often indistinguishable from other CPLs, especially MCN and BD-IPMN. Differential diagnosis is important, since SCN is a benign lesion, while MCN and IPMN have a potentially malignant behavior. In a series of 41 CPLs, significant differences in lesion morphology were found among SCN, MCN and IPMN: Macrocystic SCN has multicystic or lobulated contour with or without septation, MCN has smooth contour with or without septation, and IPMN has either a pleomorphic or a clubbed finger-like cystic shape [33]. Besides, SCN may be distinguished from BD-IPMN because the latter communicates with MPD and the communication is usually visible at MRCP.

**Fig. 6:**
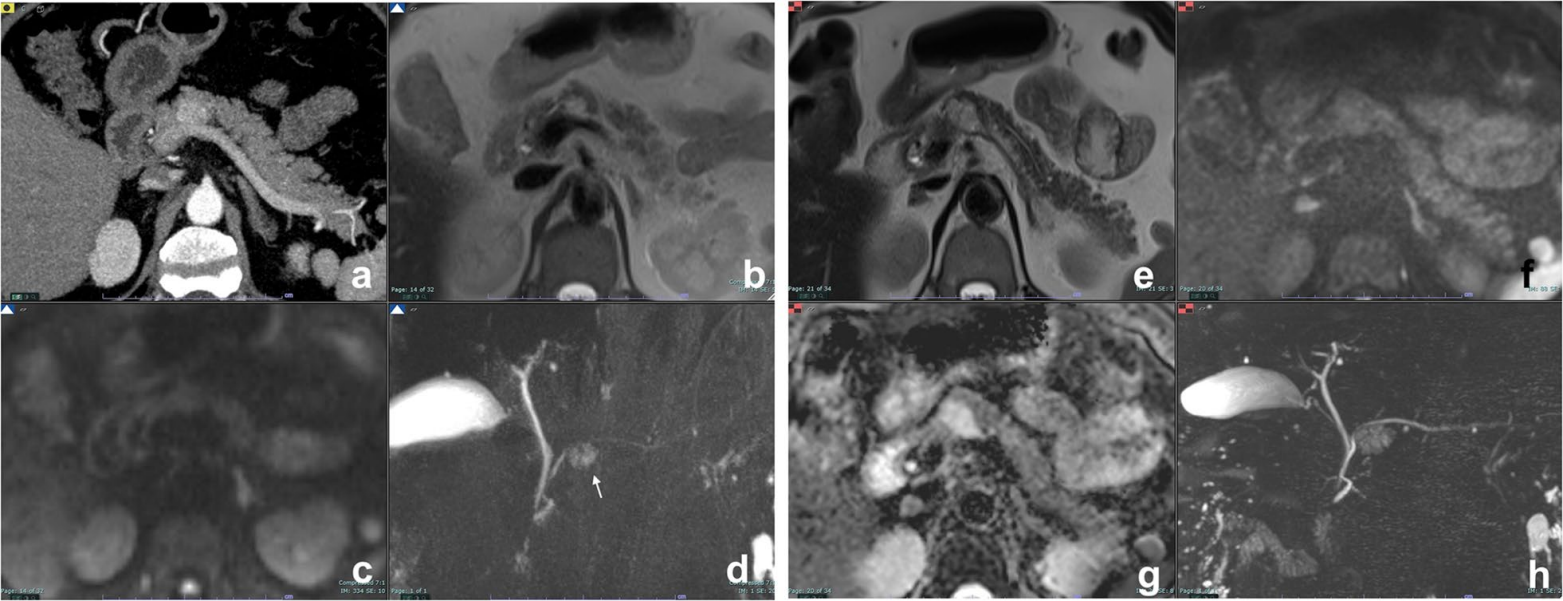
Serous cystadenoma, pseudosolid pattern. A small hypervascular lesion in the body of the pancreas is visible at contrast-enhanced CT, arterial phase (**a**). On T2w, the lesion is hyperintense (**b**), with no high signal intensity at DWI b 800 (**c**). At MRCP (**d**), the cystic components are well recognizable (arrow) and MPD is normal. **e**–**h** Follow-up at 5 years. A slight increase in size can be appreciable with no others significant changes. At ADC map (**g**), the lesion is clearly hyperintense due to cystic content

Rarely also a pseudosolid variant of SCN has been described. Microscopically, the serous solid adenoma shows architectural and cytological characteristics similar to serous microcystic cystadenoma, where the small size of cystic spaces, made by round or ovoid structure formed of cuboid cells derived from the ductular epithelium, is related to the fact that the solid adenoma has no secretory activity, giving a more compact structure compared to cystic forms. Stromal components of these lesions are characterized by avid contrast enhancement, thus misleading to an incorrect diagnosis of neuroendocrine tumor at MDCT (Fig. 6a); however, MRI, thanks to its high sensitivity to static fluids, can easily characterize this variant [34, 35] (Fig. 6).

On a multinational review on 2622 patients with SCN [26], Jais et al. observed that the SCN-related mortality was almost null, and thus, conservative treatment is suggested for these lesions.

**Fig. 7:**
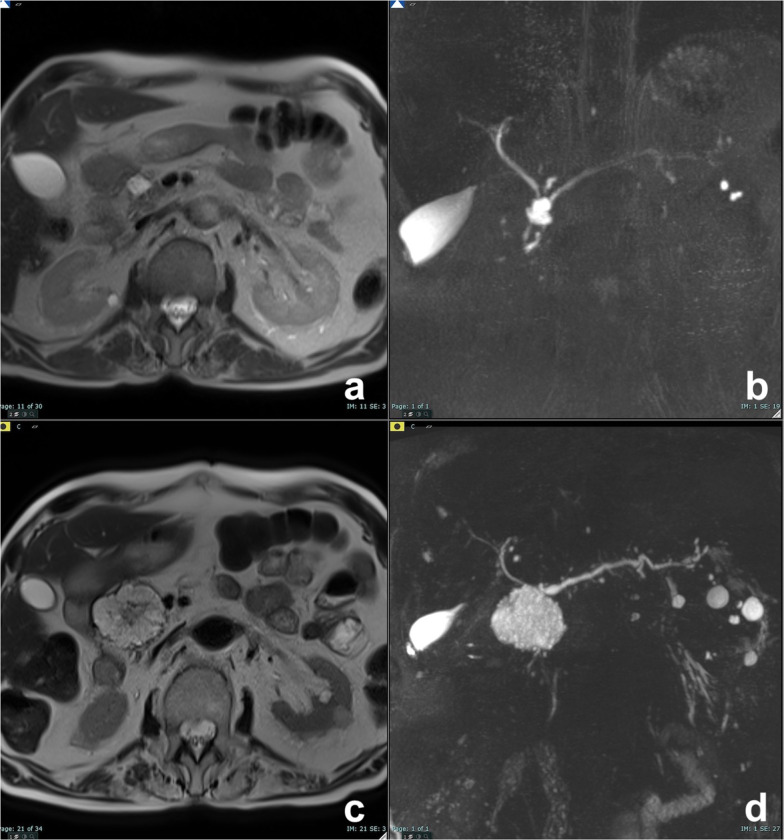
Microcystic serous cystadenoma with chronic obstructive pancreatitis. On T2w (**a**), a small lesion not clearly characterizable is appreciated in the neck of the pancreas. At MRCP (**b**), the wirsung duct is normal. **c**, **d**: Follow-up after 12 years: On T2w (**c**), the lesion is increased in size with a microcystic appearance and a large central scar, typical of a serous cystadenoma. At MRCP (**e**), a dilatation of wirsung duct with an appearance of chronic obstructive pancreatitis is clearly visible

Although recent guidelines do not recommend it [9], follow-up of SCN is suggested by some authors, as size increase is observed in nearly 40% of patients and it can lead to chronic obstructive pancreatitis (Fig. 7), thus requiring surgery [6, 26, 36].

#### Mucinous cystic neoplasm (MCN)

MCN is a cystic-forming epithelial neoplasm which accounts for 10% of CPLs and which can be classified into MCN with low–intermediate grade of dysplasia, MCN with high grade of dysplasia and MCN with an associated invasive carcinoma [37].

This lesion is composed by mucin-producing epithelial cells supported by ovarian-type stroma, and thus, it occurs almost exclusively in females (99.7%), and it is almost always located in the pancreatic body or tail (94.6%) [6, 38, 39].

The peak of incidence of MCN is in the 40 s [6]; however, it can be diagnosed in a wide range of ages with worse degree of malignancy in advanced ages; this suggests progression from benign to malignant lesions, as confirmed by the concomitant presence of different degrees of differentiation, from benign to overtly malignant, in the same lesion [6, 40].

As for SCN, MCN may manifest with unspecific symptoms such as abdominal pain and discomfort, seldom referred to the pancreatic region. Only advanced malignant lesions may manifest with more evident clinical signs such as dyspepsia, pain, weight loss and jaundice [37].

**Fig. 8:**
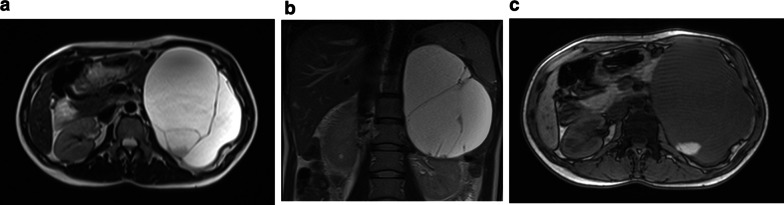
Mucinous cystadenoma, multilocular pattern. On T2w axial sequence (**a**), a large multilocular cyst is appreciable in the tail of the pancreas. The multilocular pattern is better appreciable on coronal T2w sequence (**b**). Administration of paramagnetic contrast media was avoided as patient was pregnant. On axial T1w scan (**c**), an hyperintense deposit is appreciable in the lower part of the cyst, corresponding to mucin deposit

**Fig. 9:**
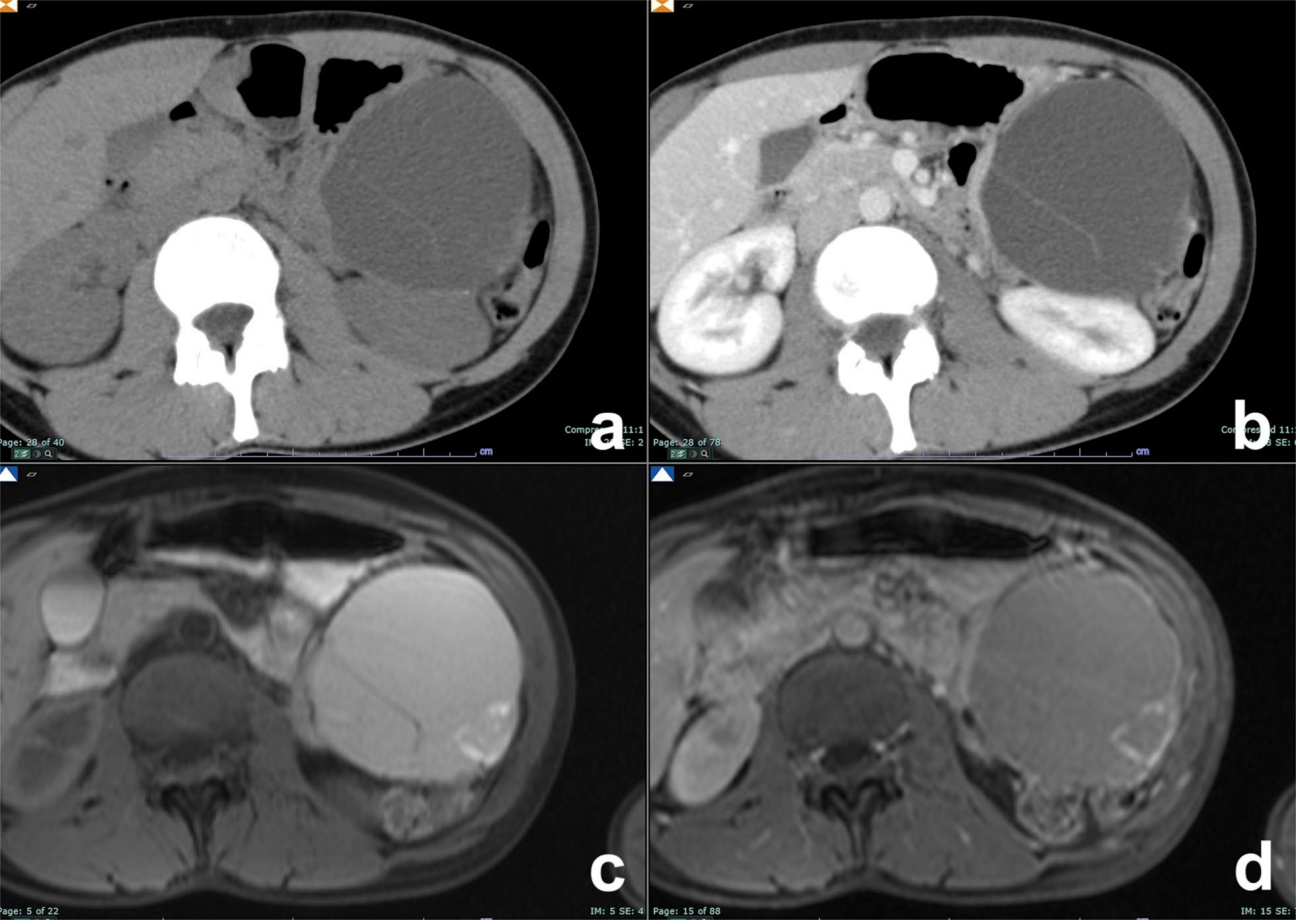
Mucinous cystadenoma, multilocular pattern. At unenhanced CT (**a**), a large cyst with a tiny septum is visible in the tail of the pancreas, which shows a slight enhancement after contrast media administration (**b**). On T1w fat-saturated GRE image (**c**), some hyperintense foci are visible in the inferolateral aspect of the cyst, which do not show significant enhancement after injection of paramagnetic contrast media (**d**), related to mucin content

At imaging, MCN appears as a round cystic masse with sharp margins whose content may have high signal intensity on T1w images due to the presence of mucin and hemorrhage (Figs. 8, 9) [37].

Two main patterns of MCN are described: macrocystic multilocular and macrocystic unilocular [37]. Macrocystic multilocular pattern (Fig. 8) is the most common and typical aspect of MCN, while MCN with macrocystic unilocular pattern may be confused with other CPLs [37], such as macrocystic SCA (Fig. 5) and pseudocyst (Fig. 1) [41]; as a matter of fact, the diagnosis of a single unilocular cystic pancreatic lesion needs correlation with clinical and with epidemiological data (previous history of acute or chronic pancreatitis, age, gender, site of the lesion) and sometimes remains undetermined, thus requiring more invasive diagnostic tools, such as endoscopic ultrasonography-guided aspiration and biopsy [42].

Radiological signs statistically associated with malignancy are: papillary vegetations, nodules, septa and wall thickness > 3 mm, size > 7 cm, number of loculations > 4, hyperintensity of cystic content on T1w images, compression and/or infiltration of adjacent vessels or organs and metastases [37, 43]. The best cutoff value to identify malignant degeneration is the presence of three of these features, with an accuracy of 91% [43].

In fit patients, resection is recommended also in MCN without radiological features of malignant degeneration, due to its high malignant potential. However, in elderly patients with comorbidities follow-up may be an option if lesion is smaller than 4 cm and if mural nodules are absent [44].

Surveillance is recommended with MRI, EUS or a combination of both every 6 months for the first year and then annually if no changes are observed [9].

#### Solid pseudopapillary neoplasm (SPN)

SPN is a rare pancreatic cystic tumor (accounting for 1–2% of all exocrine pancreatic tumors) with a low malignant potential (more than 80% SPN are benign) and favorable prognosis [45].

This tumor is solid and appears as a round, well-encapsulated mass with a variable amount of necrosis and hemorrhage, responsible of its frequent cystic appearance [45], and sometimes (29%) it may contain calcifications [46]. It usually affects young women in their 30 s and has no site predilection, even if they are more frequently located in the tail [45].

This lesion is usually discovered incidentally, as most patients with SPN are asymptomatic, but sometimes it may manifest with unspecific symptoms such as abdominal pain together with palpable mass [45, 47].

**Fig. 10:**
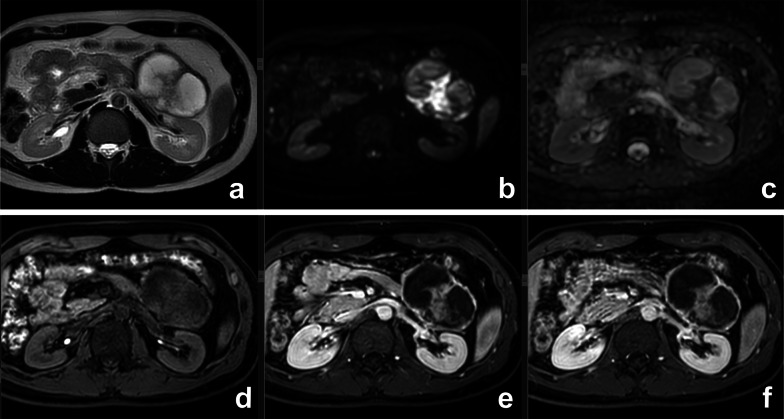
Solid pseudopapillary neoplasm. On T2w (**a**), a large cystic lesion with solid components is appreciable in the tail of the pancreas. The solid component shows high signal on DWI b 800 (**b**) and restricted diffusion at ADC map (**c**) and contrast enhancement on T1w after paramagnetic contrast media administration (**d**–**f**)

At MRI, SPN appears as a well-defined round mass, with heterogeneous signal intensity on both T1 and T2w sequences due to necrosis and hemorrhage. Solid components are typically located in the periphery of the lesion, appear well vascularized and enhance later than pancreatic parenchyma (Fig. 10) [46]. When present, acute and subacute hemorrhage is easily recognizable with high signal intensity in T1w sequences and fluid-debris level.

When large cystic changes are present (Fig. 10), SPN appears as a macrocystic uni- or multilocular lesion, similar to MCN [48]; in this situation, the younger age of the patient lays for SPN. As it usually exhibits benign behavior, it can be treated with pancreatic sparing resection [49].

#### Acinar cell cystic neoplasm (ACCN)

ACCN is a very uncommon benign cystic lesion characterized by serous content and prominent acinar cell differentiation of the epithelial lining, unrelated to the major ductal system [50].

Its exact nature is still unclear: Initially described as a non-neoplastic entity such as developmental anomaly or post-obstructive glandular dilatation and referred as “acinar cystic transformation” (ACT), subsequent studies considered it a neoplastic lesion, so further studies are necessary to clearly demonstrate dystrophic versus neoplastic origin [51].

It can manifest as unilocular and, more frequently, multilocular cyst which typically lacks solid areas of papillary projections and which can either be confined to an anatomic region or diffusely involve the entire gland [50–52].

It can occur in a wide range of ages with a striking female predominance and causes clinical symptoms in half cases, in particular abdominal pain.

**Fig. 11:**
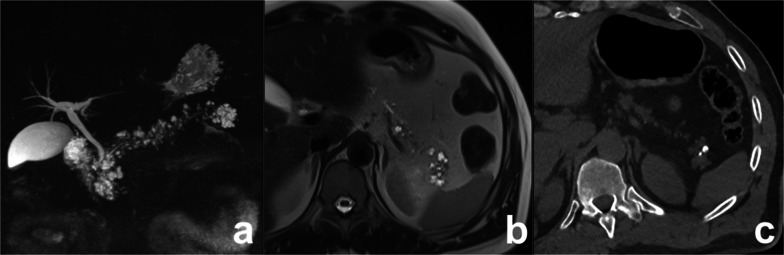
Acinar cell cystadenoma. At MRCP (**a**), there are several small cysts thought the pancreas. On T2w (**b**), some small hypointense foci are appreciable in the tail of the pancreas which at CT show up to be calcifications (**c**). At biopsy, an acinar cell cystoadenoma was found

Preoperative diagnosis is usually based on a combination of clinical features and radiological findings, but is often difficult because of similarity to other cystic pancreatic lesions. In particular, radiological findings of unilocular ACCN overlap with those of MCN and macrocystic SCN, while the main differential diagnosis of multilocular ACCN is IPMN. Delavaud et al. demonstrated that four radiological findings are significantly associated with ACCN (five or more cysts, clustered peripheral small cysts, presence of cyst calcifications and absence of communication with the main pancreatic duct) (Fig. 11) and that the presence of at least two or three of these imaging criteria had a strong diagnostic value for ACCN with a Se of 100% and 80% and a Sp of 85% and 100%, respectively [53].

As there have been no reports of malignant transformation, it is crucial to correctly characterize ACCN since it requires conservative treatment (even if pathological confirmation is still needed) [50–52].

When diagnosis of ACCN is suspected, patients should undergo surgical biopsy under laparoscopy with frozen section examination (the diagnosis of ACCN with US-guided fine needle aspiration is very difficult, since ACCN lining cells are a true mimic of normal acinar cells) and once the diagnosis is confirmed, patients must be followed up, because the exact nature of ACCN (dystrophic or neoplastic) is not clearly defined yet [53].

#### Cystic pancreatic neuroendocrine tumor (CPNET)

Cystic pancreatic neuroendocrine tumor (CPNET) is a distinctive subgroup of pancreatic neuroendocrine tumors (PNETs) with unique clinical, radiological and pathological features [54–56].

Its etiology is not clear yet. On gross pathology, it usually appears as well-demarcated lesions with centrally or eccentrically located single thin-walled cystic area containing clear fluid which does not communicate with pancreatic ducts and which is separated by a thin fibrous band from neoplastic cells.

Different authors demonstrated that compared with solid PNET, CPNET is more likely non-functioning, solitary and more likely associated with MEN1 syndrome. It is less likely to demonstrate tumor necrosis, perineurial invasion, vascular invasion, regional lymph node metastases and synchronous distant metastases and present with a lower pathological stage at diagnosis, with low Ki-67 index and mitotic count [54–56]. Moreover, patients with resected CPNET have a better 5–10-year disease-free survival compared with solid PNET [56].

It occurs over a wide range of ages, more often in adults (mean age: 53 years) and in men, and it accounts for 3.6–36.1% of resected PNETs [56].

**Fig. 12:**
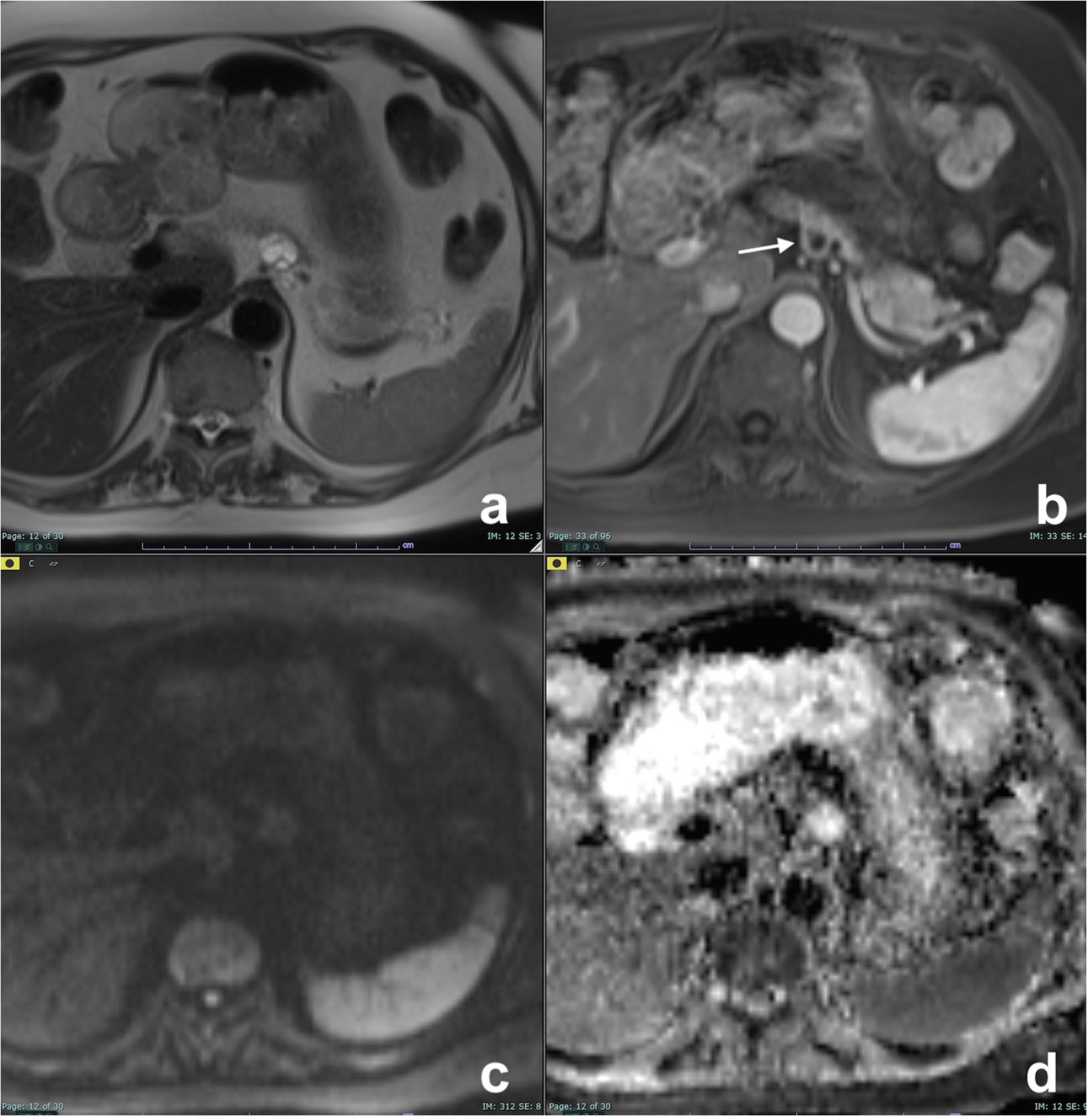
Cystic PNET. On T2w (**a**), a small hyperintense lesion with tiny septa is appreciable in the body of the pancreas. At T1w (**b**), during injection of paramagnetic contrast media an intense rim enhancement can be appreciable in the arterial phase (arrow). On DWI (**c**), no restricted diffusion can be appreciated and the lesion appears hyperintense at ADC map (**d**). After surgical removal, a G1 PNET was diagnosed at pathology

At CT or MRI, CPNET appears as non-communicating cystic lesion surrounded by a rim of well-vascularized tissue that enhances in the arterial phase [57] (Fig. 12). The main differential diagnosis is with MCN and BD-IPMN [55].

Because of its indolent behavior, more conservative surgical procedures are proposed such as tumor enucleation and spleen preserving distal pancreatectomy, and in some selected patients, particularly those with purely cystic tumor < 2 cm, only surveillance is suggested [58–60]. However, preoperative radiological diagnosis of CPNET is difficult with a misdiagnosing rate up to 50% even in high-volume centers specialized in pancreatic tumors [55, 56]; anyway, when operators are expert and when sufficient material is present for immunohistochemical staining, preoperative diagnosis can be achieved by EUS-fine needle aspiration with a diagnostic accuracy up to 100% [55–57, 61].

### CPLs communicating with MPD: IPMN

IPMN represents a large and heterogeneous group of epithelial mucin-producing tumors communicating or involving the main pancreatic duct.

It is the most common CPN, accounting for about 70% of all pancreatic cystic neoplasms, may be multifocal and have a malignant potential following an adenoma–carcinoma sequence. IPMN can display the full spectrum of histologic changes, from hyperplasia, adenoma, borderline tumor to in situ or invasive carcinoma, with a different incidence between main duct IPMN (MD-IPMN) and branch duct IPMN (BD-IPMN) [62, 63]. Moreover, it is characterized by a unique feature such as increased de novo development of pancreatic ductal adenocarcinoma (PDAC) elsewhere in the pancreas, suggesting the presence of diffuse pathologic changes predisposing to malignant transformation.

According to the cell lineage of the papillary component, four histological subtype of IPMN with different clinical pathological behavior can be distinguished: gastric, intestinal, oncocytic and pancreatobiliary [62, 64].

The gastric subtype, which accounts for the vast majority of BD-IPMNs, is typically of low grade with only a small percentage developing into carcinoma; however, if a carcinoma does develop, it is usually of the tubular type and behaves like a conventional PDAC.

A significant portion of MD-IPMN is of the intestinal type, and large and complex intestinal-type IPMNs can have invasive carcinoma, typically of the colloid type with a relatively indolent behavior.

The oncocytic type tends to be large, has relatively uncommon and limited invasion, seems to have a very good prognosis and tends to recur in the remaining pancreas years after the initial resection.

The pancreatobiliary type is the least common and is regarded as a high-grade version of the gastric type. Invasive carcinoma associated with pancreatobiliary-type IPMN is usually tubular and aggressive.

According to its site, three morphological types of IPMN have been described: the main duct type (MD-IPMN), the branch duct type (BD-IPMN) and the mixed type which meets both the criteria for MD-IPMN and for BD-IPMN. Frequencies of malignancy are significantly different according to the morphological types and have been higher for MD-IPMN/mixed type (mean: 61.6%) and lower for BD-IPMN (mean: 25.5%) [62, 64].

IPMN is diagnosed at a mean age of 60 years, and it affects males slightly more frequently than females [62, 63].

IPMN is mostly detected in asymptomatic patients, but sometimes impaired outflow of pancreatic juice induced by hypersecretion of mucin may cause pain, may induce laboratory test abnormalities of pancreatitis and may cause acute pancreatitis itself, and thus, an IPMN may be discovered after an episode of acute pancreatitis. More severe symptoms, such as jaundice, severe abdominal pain, anorexia, weight loss and diabetes, are more likely associated with malignant behavior [64].

At imaging, IPMN appears as a cystic dilatation of the involved ductal segment, caused by mucin secretion. MRI is the most sensitive technique to characterize IPMN, and in particular, MRCP is the most important sequence to demonstrate the involvement or the communication of MPD which are the key findings for the appropriate diagnosis of IPMN. Even endoscopic retrograde cholangiopancreatography (ERCP) may fail in demonstrating mucin-filled secondary duct dilatation [62].

**Fig. 13:**
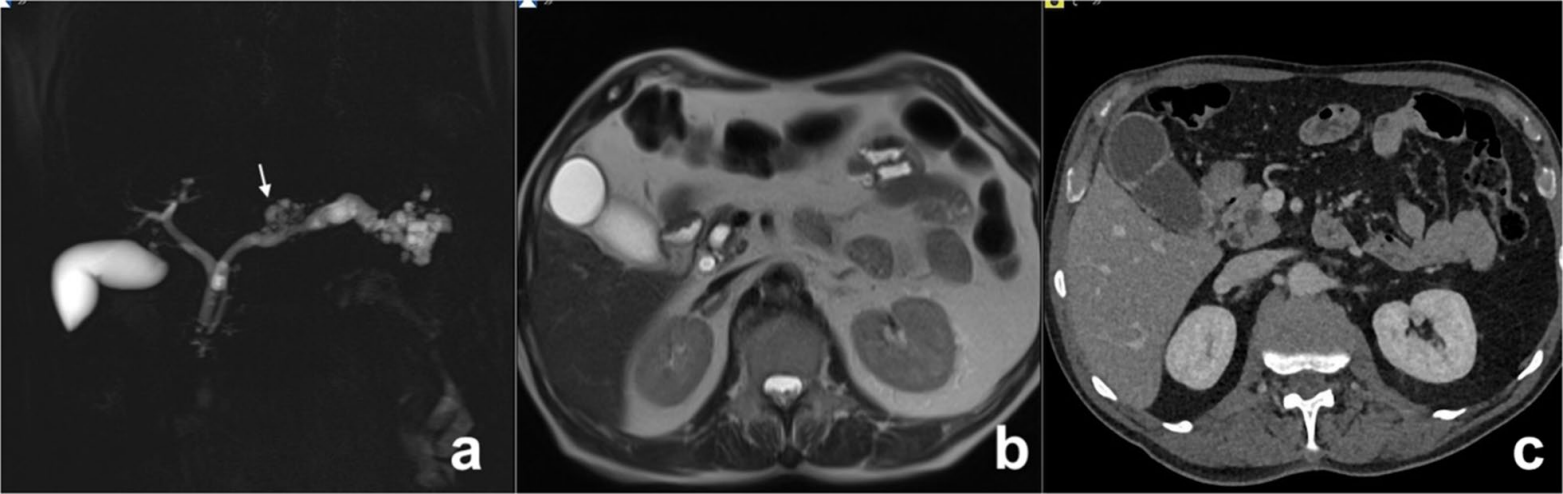
Diffuse MD-IPMN. At MRCP (**a**), a diffuse enlargement of the MPD is appreciable through the gland with a grape-like dilatation of branch ducts (arrow) and without significant causes of obstruction nor calcifications both on T2w (**b**) and CT (**c**)

**Fig. 14:**
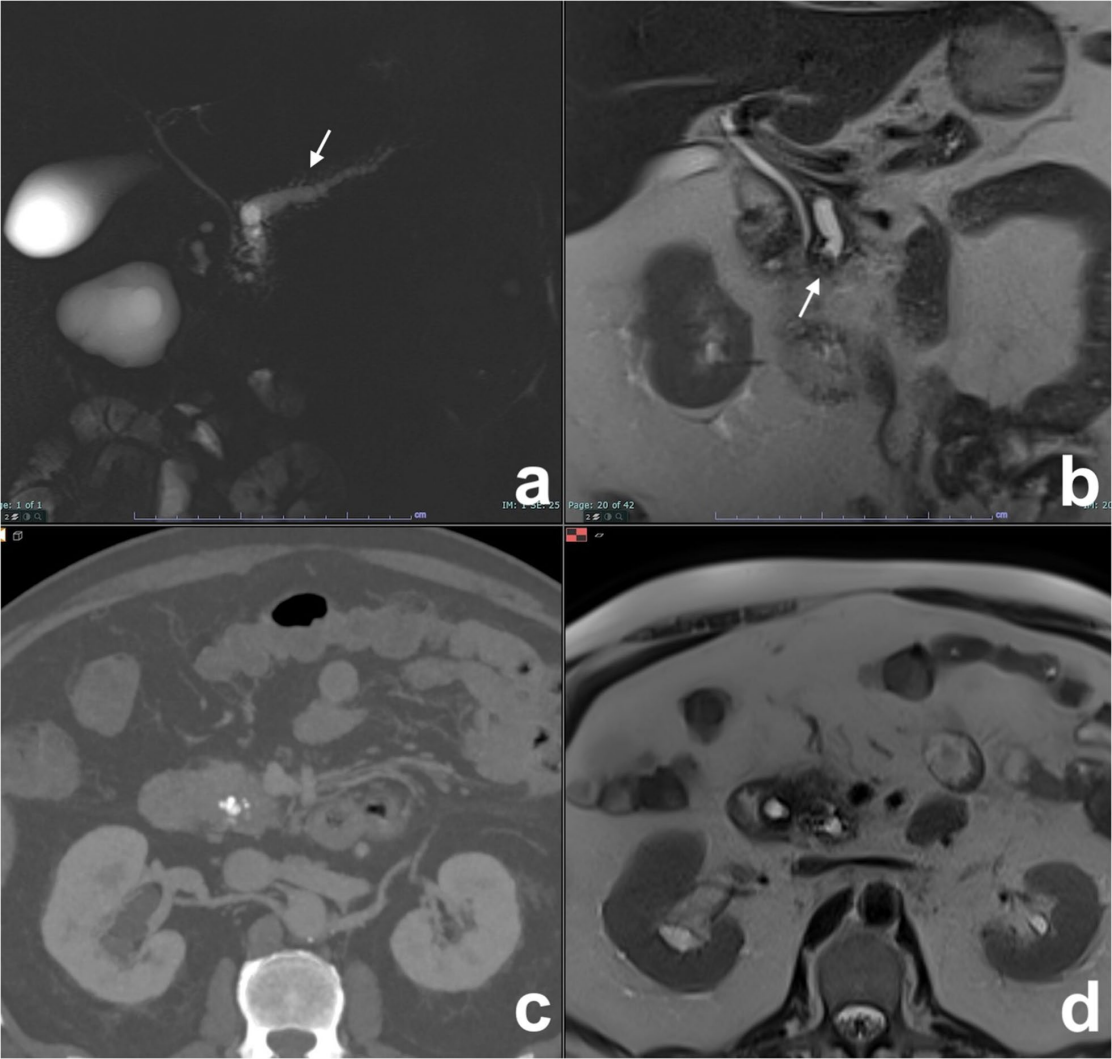
Chronic obstructive pancreatitis. At MRCP (**a**), a diffuse dilatation of the main pancreatic duct is appreciable through the gland with finger-like dilatation of branch ducts (arrow). At T2w, a defect in the prepapillary portion of the wirsung duct is appreciable (arrow). At unenhanced CT (**c**), a gross calcification is visible in the head of the pancreas, related to the intraductal calculus, responsible for the dilatation of the wirsung duct upstream, not visible on axial T2w (**d**)

MD-IPMN appears as segmental or diffuse dilatation of the MPD (> 5 mm) without other causes of obstruction [64]. In diffuse MD-IPMN, differential diagnosis with chronic pancreatitis may be difficult; however, in MD-IPMN the dilatation of MPD is more homogeneous with regular margins (Fig. 13). In a comparative study of IPMN and chronic pancreatitis, specific findings for IPMN were duct dilatation without strictures, bulging ampulla, nodule in a duct, grape-like secondary duct dilatation and nodule in a cyst, while specific findings for chronic pancreatitis were ductal dilatation with strictures, the presence of a stone and side branch ectasia with non-cystic appearance (Fig. 14) [65].

**Fig. 15:**
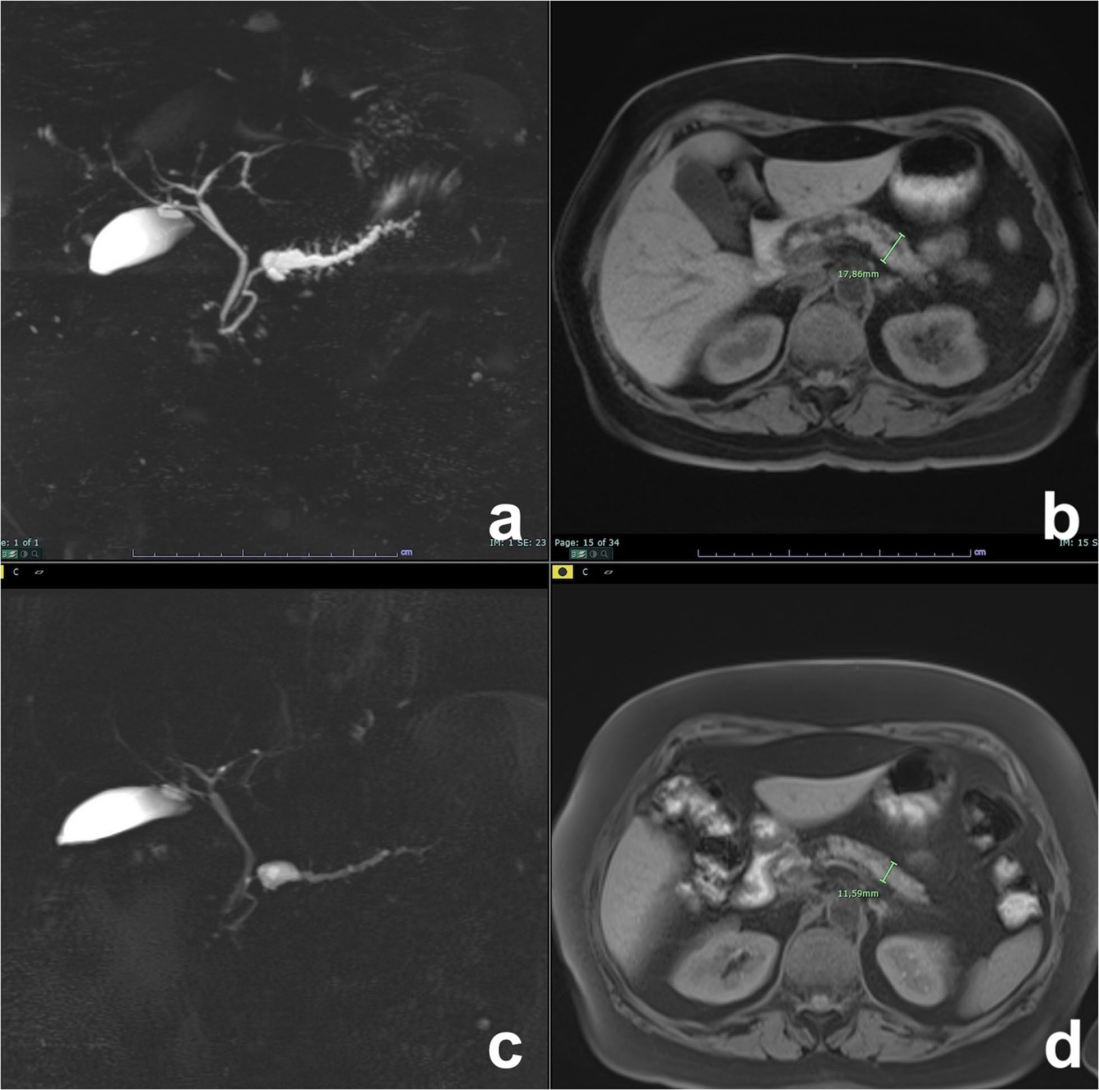
Segmental MD-IPMN. At MRCP (**a**), a segmental dilatation of the MPD is appreciable in the body of the pancreas. The MPD upstream shows signs of chronic obstructive pancreatitis (finger-like dilatation of branch ducts), but the thickness of the pancreatic gland is normal (**b**). After 20 months, the dilatation of the MPD upstream is no longer appreciable (**c**), due to atrophy of the pancreatic gland (**d**)

**Fig. 16:**
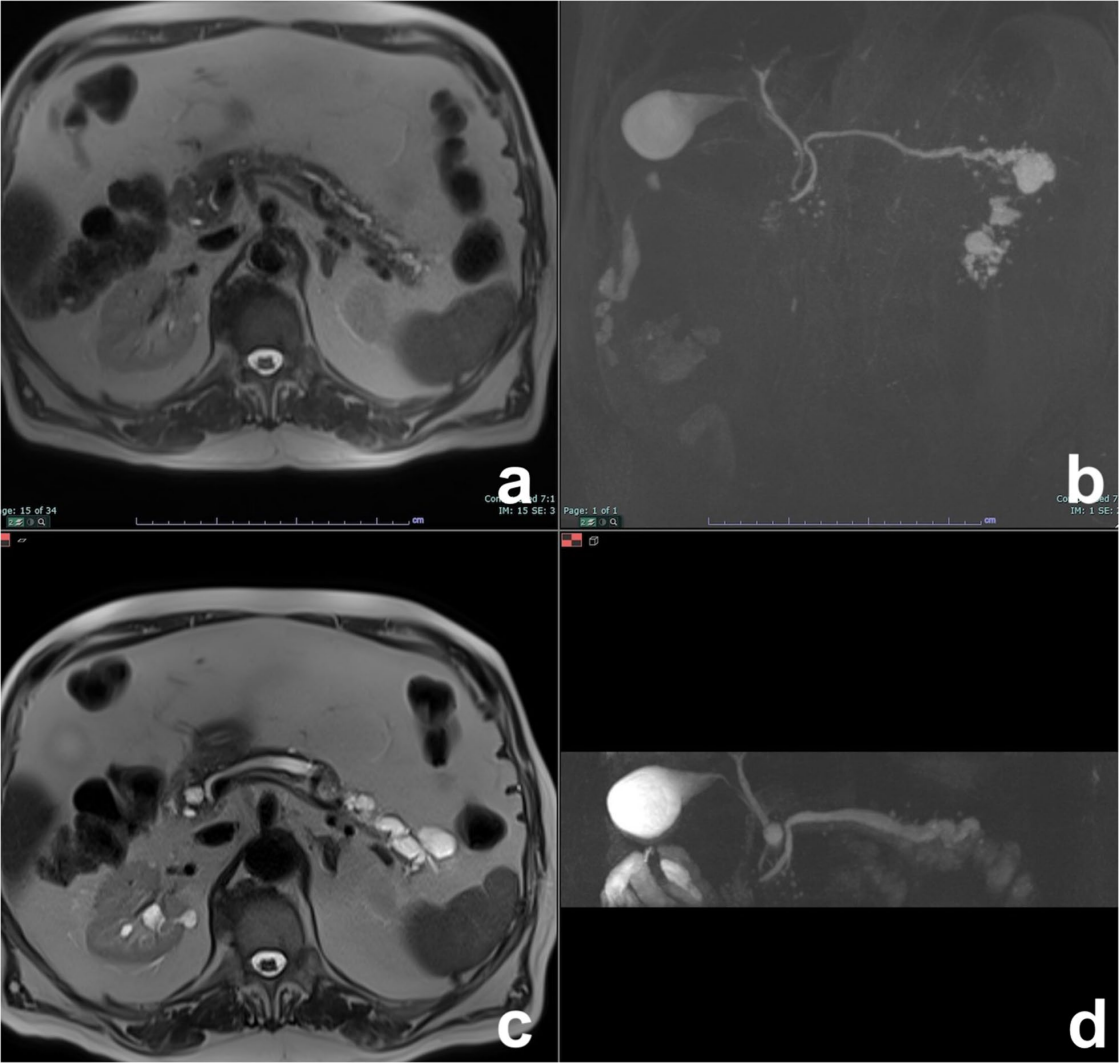
Evolution of a mixed MD-IPMN. At T2w (**a**) and MRCP (**b**), a dilatation of a branch duct in the tail of the pancreas with a slight enlargement of the MPD in the tail. After 5 years, a diffuse dilatation of the MPD is appreciable

Segmental MD-IPMN may be difficult to diagnose because it appears as a non-specific segmental dilatation without an obstruction and without a previous pancreatitis (which can lead to a post-inflammatory stenosis). The affected segment is enlarged, sometimes with dilatation of collateral ducts because of pancreatic juice outflow impairment. In these cases, also upstream dilatation of MPD may be present, thus misleading to an incorrect diagnosis of diffuse MD-IPMN (Fig. 15). In MD-IPMN, parenchyma atrophy is frequently seen (Fig. 15) [66, 67]. If left untreated segmental, MD-IPMN can grow along the main pancreatic duct till a complete involvement (Fig. 16).

Mixed-type IPMN appears as a segmental or diffuse dilatation of MPD and branch ducts along its course (Fig. 16) [68]. However, BD-IPMN may determine dilatation of MPD because of mucin overproduction but not because of MPD involvement, mimicking mixed-type IPMN.

**Fig. 17:**
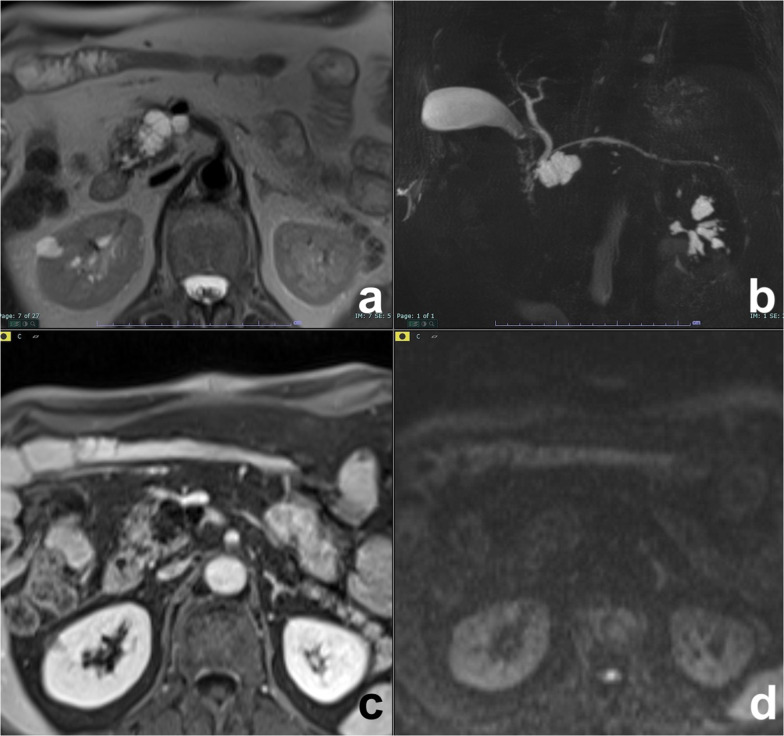
Unifocal BD-IPMN. On T2w (**a**) and MRCP (**b**), a cystic lesion with tiny septa is appreciable in the head of the pancreas. At T1w after paramagnetic contrast media injection (**c**), the septa show a slight enhancement. At DWI b 800 (**d**), no restriction diffusion can be observed

**Fig. 18:**
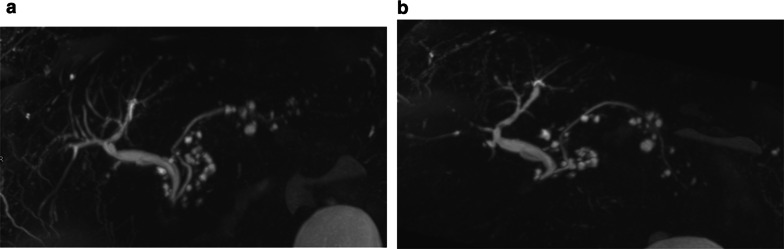
Multifocal BD-IPMN. At MRCP in coronal (**a**) and axial (**b**) projection, several round dilatations of branch ducts in thigh connection with the MPD can be appreciable

BD-IPMN appears as unifocal of multifocal cystic lesion communicating with MPD. Cysts may be uni- (Fig. 17) or multilocular (Fig. 18) with diameter ranging from few mm to some cm, are often arranged in grape-like clusters and are separated by thin septa that usually enhance after contrast medium administration [66, 67, 69]. Demonstration of communication with MPD is fundamental to make diagnosis of BD-IPMN (50), and thus, high-quality MRCP is the most sensitive and important sequence of the whole MRI protocol [70].

The main differential diagnosis of unifocal BD-IPMN is pseudocysts, MCN and SCN, since sometimes communication with MPD cannot be correctly assessed [66, 68], while the main differential diagnosis of multifocal BD-IPMN is with the rare ACCN (Fig. 11) [53].

IPMN has heterogeneous malignant potential, and thus, the International Association of Pancreatology (IAP), with Fukuoka consensus in 2012, proposed two-tier criteria to predict malignancy [64].

First tier is represented by “worrisome features,” a group of imaging findings suggesting that the lesion is not malignant yet, but could evolve in malignant, and thus requires further work-up by EUS, to better risk-stratify the lesion, and strict follow-up (Table 3).

**Table 3:** Fukuoka consensus guidelines, 2012 (intraductal papillary mucinous neoplasm—IPMN—and mucinous cystic neoplasm—MCN) and 2017 (intraductal papillary mucinous neoplasm—IPMN)

2012 Fukuoka consensus		2017 Revised Fukuoka consensus	
Worrisome features	High-risk stigmata	Worrisome features	High-risk stigmata
Cyst size > 3 cm	Obstructive jaundice	Cyst size > 3 cm	Obstructive jaundice
Thickened/enhancing cyst walls	Enhancing mural nodules	Thickened/enhancing cyst walls	Enhancing mural nodules ≥ 5 mm
MPD dilatation of 5–9 mm	MPD diameter > 10 mm	MPD dilatation of 5–9 mm	MPD diameter > 10 mm
Non-enhancing mural nodules		Enhancing mural nodules < 5 mm	
Abrupt changes in the MPD caliber with distal pancreatic atrophy		Abrupt changes in the MPD caliber with Distal pancreatic atrophy	
Lymphadenopathy		Lymphadenopathy	
History of pancreatitis		Rapid rate of cyst growth > 5 mm/2 years	
		Elevated serum level of CA 19.9	

Second tier is represented by “high-risk stigmata,” a group of imaging findings suggesting the possibility that the lesion is malignant, thus requiring surgical resection if the patient is fit (Table 3).

2012 Fukuoka consensus guidelines had been widely accepted and had high sensitivity to predict invasive carcinoma and high grade dysplasia, but relative low specificity, leading to a high number of unnecessary pancreatic resections, so in 2017 revised Fukuoka consensus has been proposed [71] (Table 3). However, this new consensus needs future cohort studies to demonstrate whether the revised criteria allow to increase specificity without jeopardizing sensitivity [63].

To date, management of IPMN is still controversial; to simplify, according to 2017 Fukuoka revised consensus, patients with IPMN with high-risk stigmata have to undergo resection if fit, patients with IPMN with worrisome features need further workup and patients with IPMN without worrisome features and without high-risk stigmata need follow-up at different intervals depending on the size of the largest cyst (Table 4).

**Table 4:** Follow-up strategies according to Fukuoka consensus guidelines

Diameter of the greatest cyst	Follow-up
< 1 cm	CT/MRI in 6 months
	Then every 2 years if no change
1–2 cm	CT/MRI in 6 months
	Yearly for 2 years
	Then lengthen interval if no change
2–3 cm	EUS in 3–6 months
	Then lengthen interval if no change alternating MRI with EUS
	Consider surgery in young fit Patients with need for prolonged survival
> 3 cm	Close surveillance alternating MRI with EUS every 3–6 months
	Strongly consider surgery in young fit patients

European guidelines distinguish absolute and relative indications for surgery (Table 5). Patients with absolute indications for surgery, patients with one or more relative indications for surgery and without significant comorbidities and patients with comorbidities and with two or more indications for surgery should be operated, while patients with significant comorbidities or short life expectancy and with only one relative indication for surgery should undergo intensive surveillance with clinical evaluation, serum CA 19.9, MRI and/or EUS every six months. Patients without indications for surgery should be followed up with clinical evaluation, serum CA 19.9, MRI and/or EUS every six months for the first year from diagnosis and then yearly [9]

**Table 5:** European guidelines criteria for resection 2018

2018 European guidelines	
Absolute indications	Relative indications
Positive cytology for malignancy/HGD	Growth rate ≥ 5 mm/year
Solid mass	Serum CA 19.9 > 37U/ml in absence of jaundice
Tumor-related jaundice	MPD diameter between 5 and 9.9
Enhancing mural nodules ≥ 5 mm	Cyst diameter ≥ 40 mm
MPD dilatation ≥ 10 mm	Enhancing mural nodule < 5 mm
	New onset of diabetes mellitus
	Acute pancreatitis caused by IPMN

Many other guidelines for management of IPMN or CPNs including IPMN have been published [24], but none of them is complete, mainly because knowledge of natural history of these tumors is not perfect yet [63].

In our opinion, the IAP guidelines, in which treatment and follow-up strategies are decided on the basis of the presence of “high-risk stigmata” and “worrisome features,” and the European guidelines are the most comprehensive and practical for decision making; however, the final decision on treatment should be individualized and should depend not only on the risk of malignancy and on the presence of symptoms, but also on patient’s life expectancy, comorbidities, operative risk, cyst location and extent of surgery.

In particular, it is important to remember that the only possible treatment is surgery, and even in high-volume centers, risk of mortality and morbidity for pancreatic surgery is high (up to 3 and 30%, respectively) [72]; moreover, according to a recent study on risks for mortality in 1800 patients with asymptomatic CPL [73], comorbidity is the major cause of mortality, and only in patients with “high-risk cysts” (considered those with size > 3 cm and main pancreatic duct dilatation) and “low-risk comorbidities,” the risk of pancreatic cancer mortality approaches that of non-pancreatic cancer mortality. Thus, patients-related factors such as age, life expectancy and comorbidities should strongly impact on clinical decision making.

All patients “fit for surgery” with high-risk stigmata should undergo surgery [64, 71], but also young patients fit for surgery with worrisome features should undergo to resection, due to their long life expectancy and higher cumulative risk of malignant progression of IPMN or de novo development of PDAC.

Patients fit for surgery without high-risk stigmata or worrisome features will undergo surveillance both for early detection of malignant progression of IPMN and for early detection of concomitant PDAC; surveillance for PDAC is needed also in patients who have undergone resection, even for noninvasive IPMN [63, 74].

The interval and period of surveillance are still controversial: IAP guidelines recommend intervals stratified by the cyst size, which can be lengthened after 2 years of stability; other guidelines recommend discontinuation after 2 years (American College of Radiology 2010) or 5 years (American Gastroenterology Association 2015) of stability [63], because, according to the authors, the small risk of malignant progression in stable cysts is outweighed by the costs of surveillance. However, according to Tanaka [63] discontinuing or lengthening surveillance may be dangerous because of the long-lasting risk of concomitant PDAC in patients with IPMN [63], and even a 6-month interval might be insufficient for an early diagnosis of PDAC [75, 76]. In particular, patients with IPMN or patients who have been resected for IPMN need follow-up not only to early detect malignant degeneration of IPMN, but also to early detect concomitant PDAC, synchronous or metachronous.

Tada et al. [77], in a study on 197 patients with IPMN and other CPLs, observed that this population is “at high risk” of developing a PDAC, with an incidence of pancreatic cancer 22.5 times higher than expected mortality in general population; Miyasaka et al. [74], on a surveillance study on 195 patients who underwent partial pancreatectomy for IPMN, demonstrated that the risk of malignant progression of IPMN and of developing a PDAC may even rise after 5 years; and He J et al. [78], on a study on a surveillance 130 patients in follow-up after pancreatic resection for IPMN, observed that the risk of developing a new IPMN requiring surgery or PDAC was 0%, 7% and 38%, respectively, at 1, 5 and 10 years.

Thus, all patients with IPMN who are fit for treatment, even those who have been resected for a noninvasive IPMN, need indefinite surveillance which should be stopped only when the patient becomes unfit for surgery. The interval of follow-up is still a matter of discussion; however, it should be kept in mind that a long (more than 1 year) interval is dangerous, as after such a long follow-up a new appearing lesion could already be unresectable at diagnosis [75].

While follow-up strategies for early detection of malignant degeneration of IPMN are well established on the basis of dimensional criteria, there are not screening criteria for early detection of concomitant PDAC yet. Different studies have demonstrated that IPMN is a major risk factor for PDAC (having PDAC 1% annual prevalence of concomitance with IPMN) [79–82], but some patients with IPMN have even higher risk of developing PDAC, in particular patients > 70 years and women [79, 82], patients with benign gastric-type IPMN without guanine nucleotide-binding protein alpha-stimulating (GNAS) mutations [82–84] and patients with IPMN and family history of PDAC especially with affected first-degree relatives [82, 85, 86]. This group of patients should be considered at higher risk, and tailored follow-up strategies should be applied.

A short interval follow-up should be considered in these categories, in order to avoid that a PDAC arising in the interval between two studies becomes unresectable, in particularly in young patients, as the cumulative risk of developing PDAC can rise up to 38% at 10 years [78]. As suggested by He et al. [78], cross-sectional imaging (MDCT or MRI) every 6 to 12 months for the first 5 years and annually thereafter should be offered.

This follow-up strategy of course rises the big issue of a large number of asymptomatic patients overloading radiological centers, due to the high prevalence of CPLs, especially in older patients (up to 40%) [82, 87]. To overcome this problem, an effort from multidisciplinary teams should be made in order to select patients who should undergo such a strict follow-up, not only on the basis of the potential malignant degeneration and de novo PDAC development, but also on the basis of patients’ comorbidities and operative risk. Moreover, as the best modality to follow these patients is MRI, and since it is “time-consuming,” fast pancreas-dedicated MRI protocols should be considered.

Some authors have already established that a short MR protocol provides information equivalent to a more time-consuming and costly comprehensive protocol in the detection of significant changes of cystic lesions as well as in the detection of mural nodules [88]. Moreover, in our experience (analysis in a series of 200 patients, data still not published) with a fast non-contrast protocol (compared with a full MR protocol with contrast media injection), it is possible to detect a small PDAC with a sensitivity and negative predictive value ranging from 92%-95% and 98%-99%, respectively.

### Endoscopic ultrasound-fine needle aspiration (EUS-FNA) and cyst fluid analysis and cyst-wall biopsy

Considering the morbidity and mortality rate of pancreatic surgery [72, 73], it should be very important to obtain correct preoperative diagnosis and correct preoperative evaluation of the grade of malignancy of CPLs.

A combination of the clinical history, gender and imaging findings sometimes is not enough in order to obtain a correct diagnosis. In such situations, EUS, EUS-FNA, EUS-TTNB can be helpful diagnostic tools: cytology, cyst fluid analyses of carcinoembryonic antigen (CEA), amylase and molecular biomarkers as well as cyst wall biopsy, allow distinction of mucinous vs non-mucinous cystic neoplasms and permit identification of the specific histological types [9, 63].

Analysis of cyst fluid obtained under EUS guidance permits the measurement of tumor markers and pancreatic enzymes, makes possible cytologic evaluation and allows to perform molecular DNA sequencing techniques which may help to distinguish serous versus mucinous cysts and to grade the epithelium of mucinous cysts [63].

Although accuracy of morphological findings of EUS in the differential diagnosis of PCLs is extremely operator dependent, ranging from 40 to 96% [89], more invasive techniques such as FNA for fluid analysis and cytology or endoscopic ultrasound-through the needle biopsy (EUS-TTNB) can increase significantly the diagnostic performance.

EUS is operator dependent and cyst fluid analysis very complex, so EUS with FNA and cytological and molecular analysis should be performed only in reference centers.

Elevated CEA allows distinction between mucinous and non-mucinous cysts (but not malignant versus non-malignant cysts), and a cutoff value of ≥ 192–200 ng/ml is 79% accurate for the diagnosis of a mucinous cyst with moderate sensitivity (73%) and specificity (84%), while a cutoff value of < 5 ng/ml is the most valuable to exclude mucinous neoplasm and suspect SCN [90, 91].

Elevated amylase levels in the cyst fluid are highly specific for pseudocyst, and a cutoff value of 250 U/L is associated with the diagnosis of pseudocyst with a sensitivity of 44% and a specificity of 98%, and thus, amylase level < 250 U/L virtually excludes the presence of a pseudocyst [90, 92].

Cytological analysis of cyst’s fluid obtained under EUS guidance has good specificity (up to 83%) for diagnosing mucinous cysts, but very low sensitivity (< 50%) because of the small amount of cells dispersed in the fluid, and moreover is difficult to interpret [91, 93–95].

Molecular analysis of the cyst fluid for diagnosis is still evolving; detection of KRAS gene mutation more accurately supports the diagnosis of a mucinous but not necessarily a malignant cyst (96% Sp) [71, 96–98], while identification of GNAS gene mutation may be helpful in distinguishing significant mucinous cysts from indolent cysts that could be conservatively managed [99, 100].

Cytology brush is not indicated because of the high rate of complications (8–20%, included death) and of technical failure (27%) with conflicting results [90, 101–103].

Tissue acquisition is the ideal method for diagnosis and risk stratification in CPLs. Barresi et al. [95], in a multicenter study on 56 Patients with CPLs, demonstrated that EUS-TTNB is a promising technique that provides specimens adequate for histological diagnosis in 83.9% of cases with acceptable rate of adverse events (16%), all of which classified as mild.

### New imaging applications

Distinguishing invasive from noninvasive IPMNs preoperatively remains challenging. Khoury et al. in a cohort of 478 pancreatectomies performed for IPMN in a single year in over 100 US institutions found that invasive carcinoma or high-grade dysplasia was identified only in 23% of resected lesions, so improved biomarkers are needed to aid in surgical selection [104].

In the last years, different studies have demonstrated that quantitative imaging and radiomics applied to MDCT pancreatic protocols can provide markers for reliably discriminating benign from malignant IPMNs (low- and intermediate-grade dysplasia versus high-grade dysplasia and invasive carcinoma), thus allowing objective risk stratification of these lesions [105–108]. In particular, in the most robust of these studies conducted on MDCT images of 408 resected patients with IPMN, Tobaly et al. [108] found that in the training cohort, 85 radiomics features were significantly different between patients with benign and malignant IPMNs; they also demonstrated that the multivariate model of radiomics only differentiated benign from malignant tumors in training cohort with an area under the ROC curve (AUC) of 0.84, Se of 0.82, Sp of 0.74 and in the external validation cohort with an AUC of 0.71, Se of 0.69 and Sp of 0.57.

MRI-based radiomics is much more difficult because MRI suffers from less standardization than CT with a large variety in the multiple parameters related to scanner properties, acquisition settings and image processing that could hamper radiomics analysis [108]. However, in a retrospective series of 248 patients with surgically confirmed IPMN, Jeon et al. among texture variables found 7 significant predictors of malignancy (*p* < 0.05), 2 of which confirmed to be significant predictors of malignancy also at multivariate analysis. Moreover, when adding texture variable to MRI findings, diagnostic performance for predicting malignant IPMN improved from 0.80 and 0.78 to 0.85 in both reviewers (*p* < 0.05) [109].

## Conclusions

CPLs are a heterogeneous group of frequently detected lesions with different malignant potential and different prognosis, requiring different therapeutic and follow-up strategies; therefore, it is fundamental to correctly characterize them. MRI with MRCP sequence is the most useful technique to detect imaging features helpful for lesion characterization.

Differential diagnosis of these cysts is based on the presence of communication or not with MPD, on their morphological features, on their location as well as on the basis of clinical history and demographic data.

Once pseudocyst and WON have been excluded, a CPL communicating with MPD is an IPMN, while if not communicating could be a SCN, a MCN or a SPN and differential diagnosis, certain or presumptive, can be done on the basis of demographic data, location of the lesion and its morphological appearance.

In some cases, differential diagnosis is still challenging and may require EUS-FNA and/or cyst-wall biopsy.

MCNs and IPMN with high-risk stigmata must be resected if patients are fit for surgery; in patients with long life expectancy and IPMN with worrisome features, resection is suggested.

Patients with IPMN without high-risk stigmata and patients who have been resected for an IPMN need follow-up till they are fit for surgery, in order to early detect malignant degeneration or concomitant pancreatic ductal adenocarcinoma.

When the diagnosis of a CPL remains indeterminate, in our opinion it should be managed as an IPMN, as they are the most frequent CPLs with a potential malignant behavior.

New imaging applications, with quantitative analysis thanks to texture analysis, have the potential to help to better stratify IPMNs, thus allowing a more correct management.

## Data Availability

Data sharing is not applicable to this article as no datasets were generated or analyzed during the current study.

## Abbreviations

ACCN: Acinar cell cystic neoplasm
CEA: Carcinoembryonic antigen
CPLs: Cystic pancreatic lesions
CPN: Cystic pancreatic neoplasm
CPNETs: Cystic pancreatic neuroendocrine tumors
ERCP: Endoscopic retrograde cholangiopancreatography
EUS: Endoscopic ultrasound
EUS-FNA: Endoscopic ultrasound-fine needle aspiration
EUS-TTNB: Endoscopic ultrasound-through the needle biopsy
GNAS: Guanine nucleotide binding protein, alpha stimulating
IPMN: Intraductal papillary mucinous neoplasm
MCN: Mucinous cystic neoplasm
MDCT: Multidetector computed tomography
MPD: Main pancreatic duct
MPR: Multiplanar reconstruction
MRCP: Magnetic resonance cholangiopancreatography
MRI: Magnetic resonance imaging
PDAC: Pancreatic ductal adenocarcinoma
SCN: Serous cystic neoplasm
SPN: Solid pseudopapillary neoplasm
US: Ultrasound
WON: Walled-off necrosis
